# Strategies to integrate physiotherapists into primary health care in South Africa

**DOI:** 10.4102/sajp.v79i1.1796

**Published:** 2023-03-14

**Authors:** Sholena Narain, Desmond Mathye

**Affiliations:** 1 Department of Physiotherapy, Faculty of Health Sciences, Sefako Makgatho Health Sciences University, Medunsa, South Africa

**Keywords:** physiotherapy, primary health care (PHC), universal health coverage, access to healthcare, National Health Insurance (NHI)

## Abstract

**Background:**

Health services are inaccessible in low-income countries. The National Health Insurance (NHI) bill, linked to primary health care (PHC), was introduced in South Africa to improve access to health services. Physiotherapists contribute to healthcare and improve individuals’ health status across their lifespan. The South African healthcare system has many challenges: physiotherapists mostly practising at secondary and tertiary levels of care; a shortage of physiotherapists in the public health systems and rural areas; the omission of physiotherapy in health policies.

**Objectives:**

To explore strategies to integrate physiotherapy services in PHC settings in South Africa.

**Method:**

Our study used a qualitative, exploratory and descriptive approach to collect data from nine doctorate physiotherapists at South African universities. Data were thematically coded.

**Results:**

The themes are to (1) improve societal knowledge of physiotherapy, (2) ensure policy representation of the profession, (3) transform physiotherapy education, (4) broaden the role of physiotherapy, (5) eradicate professional hierarchy and (6) increase the physiotherapy workforce.

**Conclusion:**

Physiotherapy is not well known in South Africa. Physiotherapy is needed to feature in health policies to transform education focussing on disease prevention, health promotion and functioning in PHC. Broadening physiotherapy roles should consider the regulator’s ethical rules. Physiotherapists should proactively collaborate with other health professionals to dismantle professional hierarchies. Without addressing the urban-rural, private-public divide, the physiotherapy workforce cannot improve, to the detriment of PHC.

**Clinical implication:**

Implementing the suggested strategies may facilitate physiotherapy integration into PHC in South Africa.

## Introduction

Health services are inaccessible to many people, particularly in poor and low-income countries (Sanogo, Fantaye & Yaya 2019). Physiotherapy is one among them (Kamenov et al. 2019). Following the United Nations Sustainable Development Summit in 2015, over 75 countries created or enacted laws to ensure universal health care access (Feigl & Ding 2013). African countries such as Kenya and South Africa have fallen behind in enacting universal health access legislation or laws to enable equitable health access for all citizens (World Health Organization [WHO] 2020). South Africa and Kenya developed health policies and bills to expand health services coverage in their respective countries (WHO 2020) in 2012 and 2018, respectively. The Kenyan health policy is called Universal Health Coverage and the South African bill is called National Health Insurance (NHI) (WHO 2020:6).

The proposed end goal of NHI is the affordability and accessibility of health services in South Africa (NHI 2019). Section 7(2) (b) of the NHI bill prescribes that access to all health services (including physiotherapy) should be at a primary health care (PHC) level (NHI 2019).

The WHO sees PHC as a vehicle for ensuring universal healthcare access to all citizens within a country (WHO 2020:4). The PHC is an accelerator to ensure healthy living and promote well-being for persons across their life span (WHO 2020a). The three main components of PHC are (1) primary care, which is a key part of the health system that supports first-contact, accessible, continuous, comprehensive and coordinated patient-focused care (WHO & UNICEF 2018), (2) multi-sectoral policy and action and (3) empowered people and communities (WHO & UNICEF 2020).

There seem to be multiple challenges within the South African health system that negatively affect access to health services (Pauw 2022) such as physiotherapy. As far as physiotherapy is concerned, these challenges include, amongst others, (1) physiotherapists mostly practising at secondary and tertiary levels of care, (2) a shortage of physiotherapists within the health care workforce, (3) unequal workforce distribution between public and private health systems, (4) unequal workforce distribution between rural and urban areas as well as (5) the omission of physiotherapy within the South African health policies.

One of the requirements of the NHI bill is that access to all health services (including physiotherapy) in South Africa shall be at a PHC level. Any services required beyond that level should be ‘referred to secondary or tertiary levels of care’ (NHI 2019). In western countries (Maharaj et al. 2018) as well as some African countries such as Nigeria (Chigbo et al. 2015), physiotherapists mainly function at secondary and tertiary levels of care, making it difficult for most citizens to access such services and the same is seen in South Africa. Apart from practising predominantly at the secondary and tertiary levels of care (Maharaj et al. 2018), physiotherapists are focused more on curative care rather than health promotion and prevention, as reported by Wendimagegn and Bezuidenhout (2019).

The health workforce shortage is a global problem cutting across various disciplines (Boniol et al. 2022). The broader discipline of rehabilitation professions, including physiotherapy, is not spared from such shortage (Al Imam et al. 2022; Hartman, Johnson & Holder 2021; Morris et al. 2021). As of April 2020, there were 8053 registered physiotherapists in South Africa, servicing about 60 million people (Health Professions Council of South Africa [HPCSA] 2021).

In 2010, approximately 82.5% of physiotherapists worked in the private sector, with only 17.5% in the public sector, creating a severely unequal workforce distribution (Van Rensburg 2014). A study by Tiwari, Ned, and Chilte (2020) showed that public services employed only 1504 physiotherapists in all nine provinces of South Africa. The number referred by Tiwari et al. (2020) includes all physiotherapists employed as per the *South African Public Services Act of 1994*, particularly, in the Health and Basic Education departments.

Additionally, there is an unequal urban–rural distribution of the workforce, with most physiotherapists concentrated in highly urbanised South African provinces as compared with rural provinces (Van Rensburg 2014). The workforce shortage, compounded with the unequal distribution of physiotherapists along public-private and rural-urban lines, creates significant challenges for communities to access physiotherapy services.

Rehabilitation has not been a priority for the South African Government (Morris et al. 2021). As such, health policy documents indicate a persistent marginalisation of physiotherapy from PHC and an apparent lack of awareness of the profession’s value in that setting (Department of Health [DOH] 2015). As an example, the NHI pilot study indicated that medical practitioners (doctors) and nurses are the key role players in the PHC setting (DOH 2015), excluding other health professions like physiotherapy (Premji & Hatfield 2016).

It is commonly accepted that physiotherapists are valuable members of multidisciplinary teams, making an essential contribution to health care through health promotion, prevention of injuries and disease, screening, triage, health assessment and treatment, and the improvement of the health status of individuals across the lifespan (Kumar 2013). Besides the roles highlighted by Kumar (2013), other researchers advocate for physiotherapists to be visible and play a meaningful role within the PHC setting (Guilcher 2018). Our study seeks to explore strategies that may be used to integrate physiotherapy services into PHC settings in South Africa to improve access in anticipation of the proposed NHI.

## Method

Participants were asked the research question: ‘What role do you think physiotherapists should be playing within the NHI?’. From participants’ responses, strategies to integrate physiotherapy in PHC emerged. The authors used a qualitative, exploratory and descriptive design to collect data from a purposefully selected sample of physiotherapy lecturers. An exploratory and descriptive design was chosen as the study sought to document and describe phenomena of interest (Marshall & Rossman 2016), which is little understood or vague (Polit & Beck 2012). The interviews were virtually conducted (via telephone and Skype) with physiotherapy lecturers from various universities in South Africa.

The authors browsed through the web pages of the eight South African university physiotherapy departments, looking at the lecturers’ qualifications and areas of research interest. These lecturers had to be (1) registered with the HPCSA and (2) hold a doctoral degree (PhD or equivalent) within the areas of public/community health or have publications in public health-related topics. These criteria were considered adequate to classify the participants as knowledgeable about the studied phenomena.

The study sample comprised nine physiotherapy lecturers. Participants were employees of local universities with positions ranging from lecturer to professor. Three participants were based at the University of Cape Town, two at the University of Limpopo and one each at the University of KwaZulu-Natal, the University of the Witwatersrand, Stellenbosch University and North-West University. The University of Limpopo and North-West University did not offer degrees in physiotherapy, but the participating lecturers were physiotherapy researchers.

The participants’ academic experience ranged from 9 to 36 years, with a mean of 16.9 years (standard deviation [s.d.] = 8.04). The participants also had work experience in the public health sector ranging from 1 to 16 years, with a mean of 7 years. (s.d. = 5.61). The private health sector experience ranged from 0 to 16 years, with a mean of 5.33 years. (s.d. = 5.39). The number of peer-reviewed journal publications ranged from 11 to 94, with a mean of 35.33 publications (s.d. = 27.51). Other demographic characteristics are included in Table 1.

**TABLE 1:** Demographic characteristics of the participants.

Code	Age	Gender	Fields of expertise
Exp 1	42	Male	Community, public health
Exp 2	42	Female	Paediatrics, health advocacy, critical care and pulmonology
Exp 3	36	Female	Motor control disorders in children
Exp 4	55	Male	Cardio-respiratory, society and disability
Exp 5	37	Female	Public health, HIV, disability, community-based rehabilitation
Exp 6	48	Female	Health promotion
Exp 7	64	Female	Research methodology, health-related quality of life and epidemiology
Exp 8	55	Female	Disability and rehabilitation PHC
Exp 9	44	Female	Adult neurological rehabilitation

HIV, human immunodeficiency virus; PHC, primary health care.

Data were collected using semi-structured interviews. A demographic data form was used to obtain participants’ demographic information. The nine participants were invited to participate via email. Upon receiving informed consent, a suitable time for the interviews was arranged. Participants were allowed to choose between telephonic or Skype^®^ interviews, and eight chose telephone, with one choosing Skype^®^. Interviews were in English and recorded.

## Data analysis

Data were analysed using a thematic approach (Green & Thorogood 2014). Data were transcribed verbatim. The first author verified the transcripts for accuracy and uploaded them into Nvivo^®^ Version 10 for coding. Codes were grouped until themes were identified.

## Trustworthiness

According to Guba and Lincoln (1981), credibility, dependability, confirmability and transferability are used to show a study’s rigour (Guba & Lincoln 1981). The authors used a well-established methodology, peer review and existing literature to gauge the degree to which the study results are congruent with the results from the latest studies to ensure credibility. Dependability was ensured by a detailed data collection and analysis process. Furthermore, the authors used an audit trail of data collection and analysis to demonstrate confirmability. Transferability was ensured by a thorough presentation of the methodology to allow the reader to find the significance of our study in studies of a similar context.

## Results and discussion

Six themes emerged from the data as strategies to integrate physiotherapy at PHC in South Africa. The codes and quotations that led to the themes are presented and discussed in this section. The themes are to (1) improve societal knowledge of physiotherapy, (2) ensure policy representation of the profession, (3) transform physiotherapy education, (4) broaden the role of physiotherapy, (5) eradicate professional hierarchy and (6) increase the physiotherapy workforce.

### Theme 1: Improve societal knowledge of physiotherapy

Available physiotherapy services that are not known by the population will likely not be utilised. Several studies have suggested a positive link or relationship between health literacy, such as knowledge of physiotherapy and its utilisation (Beales, Mitchell & Holthouse 2021; Eyinda, Myezwa & Sekome 2022; Taj-Din 2021). In our study, the limited health literacy of communities within South Africa was highlighted by participants:

‘People in poorer communities are mostly uneducated, and they will not even have a clue what physiotherapy is all about’. (Exp 1, 42 years old, male)

‘Under-resourced, undeveloped communities more often lack the knowledge or empowerment or skills to access health care … large sectors of society still do not have access to physiotherapy’. (Exp 3, 36 years old, female)

Various factors have been cited in the literature as possible reasons leading to a lack of awareness or knowledge about the profession of physiotherapy. Mbambo (2004) suggested that the South African segregation laws of the past were responsible for physiotherapy not being well known in black communities and that black communities perceived physiotherapy to be an elite profession practised by white people for white people. Furthermore, people living in urban areas were found in another study to have better awareness and knowledge of physiotherapy when compared to rural people (Shruti et al. 2022). Poor knowledge and awareness of physiotherapy are confined to lay persons and other health care professionals (Bolarinde, Omoniyi & Joseph 2021), who have a duty to refer patients for physiotherapy.

The availing of physiotherapy services at PHC without improving the awareness and knowledge about the profession and its role in the general public and other health professionals will not necessarily improve access, as utilisation will be low.

### Theme 2: Policy representation of the profession

It seems from the physiotherapy lecturers that the root cause of physiotherapy not featuring in health policy documents is that the profession’s role is not known to the policymakers. They argue that if policymakers were aware of the role and value of physiotherapy, they would have ensured that services are available at all levels of health care, including in a PHC setting (especially in rural areas). The participants suggest that physiotherapists must research the profession’s role and ensure that the existing body of knowledge or literature influences policy as per the following quotations:

‘Physiotherapy should be involved at every level of any health service. Its omission is very concerning because there just has not been the awareness that physiotherapy has an integral role to play as part of the team at all levels of health care’. (Exp 2, 42 years old, female)

‘We are supposed to be playing a consultative role and advise the government about our profession’. (Exp 4, 55 years old, male)

‘I would like physios to be more involved on a policy level. If we do not participate in the draft or the implementation stage, and our voice is missing from the beginning, it is going to be very evident when it comes to the implementation of the end-product of policies’. (Exp 5, 37 years old, female)

‘I see no reason why they should not be researchers within PHC to gather data, plan the intervention and maximise the use of resources’. (Exp 7, 64 years old, female)

In the 21st century, the role and value of physiotherapy are available in the literature. However, there is a possibility that policymakers are unaware of the available research information or physiotherapists themselves are failing to translate their research findings into policy and practice (Pascal & Joseph 2020); see quotation below:

‘We have to make a stronger case in presenting our information to policymakers by packaging it in a way that is translatable. Researching topics that are relevant to society and then getting the best evidence of care … The wealth of information that can be evaluated and assessed through ongoing monitoring of services are areas that can be researched. We must collect data for PHC’. (Exp 3, 36 years old, female)

Policymakers, by their nature, are bureaucrats and may not translate research into practice and policy. Until physiotherapy researchers reconsider ‘preaching to the converted’, where they disseminate findings to colleagues through conferences and peer-reviewed journal publications and not the general public (Kennedy, Jensen & Verbeke 2018), the status quo will likely remain. Researchers must disseminate their findings through other channels to accommodate those not scientifically converted, such as politicians, bureaucrats and the general public.

Besides influencing policy through research, participants felt the Health Profession Council of South Africa’s Professional Board of Physiotherapy, Podiatry and Biokinetics (PPB) had to be consulted whenever policymakers were in doubt (see quotation below):

‘Nobody is making sense of the data that the nurses are collecting in the clinics and hospitals on a daily basis; Physios can look at those results; that is something that is not happening but can be tapped into … Policymakers missed the impact that physio actually has on primary health care, primarily because they did not consult us. The Physiotherapy Board are the custodian of our profession. Policymakers must consult with the Board. That consultation is important because it informs you of how you should treat physiotherapy’. (Exp 6, 48 years old, female)

The PPB regulates the profession of physiotherapy in the country and is empowered in terms of section 3 of the *Health Professions Act* of 1974:

[*T*]o determine strategic policy and to make decisions concerning matters of education, training, registration, ethics and professional conduct, disciplinary procedure, the scope of the professions and maintenance of professional competence.

The regulator has direct access to the Minister of Health and is also empowered in terms of section 3 (h) of the *Health Professions Act* of 1974:

[*T*]o advise the Minister on any matter falling within the scope of the Act to support the universal norms and values of health professions, with greater emphasis on professional practice, democracy, transparency, equity, accessibility, and community involvement.

This section of the *Health Professions Act* implies that the regulator (or PPB) is statutorily empowered to influence health policies in the country.

### Theme 3: Transform physiotherapy education

While PHC requires a strong involvement in health promotion, prevention, community involvement and addressing societal needs, participants suggested that physiotherapy education in South Africa promotes services at a curative level, not PHC. As per the quotations below, participants suggest a transformation of physiotherapy education:

‘Our curriculum in the eight schools of physiotherapy in the country needs to respond to the landscape that is forever changing, so we need to prepare our graduates on public health matters, law, and ethics’. (Exp 1, 42 years old, male)

‘Make sure that we train health care professionals that can address societal needs to be stronger in their leadership, advocacy, management, and communication. That will help to bring that role across more strongly where people can recognise the value of what physiotherapists bring’. (Exp 3, 36 years old, female)

Mbambo (2004) has suggested that physiotherapists practice predominantly at the secondary and tertiary levels of care, and student training at academic institutions worldwide is based on western methods and approaches, equipping physiotherapists with skills to practice primarily in secondary and tertiary settings but not as much so for PHC settings. However, a study by Blose et al. (2019) has suggested that community-based PHC training for physiotherapy students is one of the best approaches to incorporating competencies required for practice as future independent practitioners. To ensure that physiotherapists have the adequate core competencies to practice on a PHC level, there needs to be a transformation of physiotherapy education in South Africa to focus more on health promotion and prevention as well as on addressing societal needs. The White Paper on the transformation of health in South Africa (DOH 1997) calls for the training of health professionals to shift towards disease prevention and health promotion. However, many physiotherapy training programmes have made minimal changes to their curricula (Mokwena & Phetlhe 2015; Naidoo et al. 2018).

The Lancet Commission also ‘highlights a call from 20 professional and academic leaders for significant reform in the training of doctors and other healthcare professionals for the 21st century’ (Frenk et al. 2010). It emphasises that ‘changes are needed because of fragmented, outdated, and static curricula that produce ill-equipped graduates and calls for a medical education system to produce competency-led curricula for the future’ (Frenk et al. 2010). Involvement in PHC will require a different way of working and a different set of skills. As such, physiotherapy education and skills must be appraised for physiotherapists to meaningfully participate in the contracts and services developed in the NHI (Blose et al. 2019).

Primary health care requires a diverse set of skills. As such, it is important to increase the health prevention and promotion role and ensure community participation is built into physiotherapy interventions and coded and remunerated by funders. Mostert-Wentzel, Frantz & Van Rooijen (2013) found that most of the universities’ physiotherapy curricula in South Africa address the social determinants of health, with less than half of the universities addressing community development and social responsibility.

### Theme 4: Broaden the role of physiotherapy

Participants also suggested that physiotherapy should be broadened through task shifting and role release to intensify health promotion and prevention (see quotations below):

‘We have a huge role in health promotion and prevention … Physiotherapy can actually be broadened to increase right to access healthcare in primary healthcare facilities so most people can gain access to physiotherapy services at that level’. (Exp 1, 42 years old, male)

‘For South African communities, especially rural communities with limited services, we should broaden our scope more toward the complete primary health care philosophy. When I say primary health care philosophy, I mean promotion, prevention, cure, rehab … Unfortunately, the majority of physiotherapists tend to be very much medically orientated and focus very much on one-on-one curative or impairment-based treatments … Task shifting and sharing is something that the literature is very strong on, especially in early childhood development … It is one way that we can make things a little easier regarding the load that people carry’. (Exp 8, 55 years old, female)

‘We must be involved in task-shifting models; we need to develop working strategies where we can use these task-shifting models in primary health care, we can train laypersons … Physios can train task shifters and mid-level workers to do basic assessment and management in the community and then give feedback to the qualified physios’. (Exp 5, 37 years old, female)

‘Physios can be involved in the training of physiotherapy assistants, rehab assistants, carers, and community health care workers to provide rehabilitation at home to reduce the burden on the healthcare facility’. (Exp 2, 42 years old, female)

‘Physios have an important role in training home-based carers, ancillary workers, and mid-level workers, monitoring their performance, interacting with them, and also learning from them’. (Exp 7, 64 years old, female)

‘We have a role in preventing injuries in the community through health promotion and education based on the cause and nature of diseases’. (Exp 4, 55 years old, male)

According to Mandeville et al. (2016), sub-Saharan Africa has the acutest shortfall of health care workers. Task shifting has been endorsed as one response to the global health worker crisis. Lekoubou et al. (2010) describe task-shifting as:

[*A*] situation where a task normally performed by a physician is transferred to a health professional with a different or lower level of education and training, or to a person specifically trained to perform a limited task only, without having formal health education.

Seidman and Atun (2017) reported that task shifting presents a viable option for decreasing costs and improving health system efficiency in low- to middle-income countries. However, the authors recommended future research to investigate whether task shifting can reduce expenses for activities related to emerging global health priorities. Crowley and Mayers (2015) also agree that task-shifting is a crucial strategy for governing human resources for health care, and recommended that African governments review current task-shifting programmes to include a broader range of programmes and initiatives to address the current challenges. However, there is a limit to how far task shifting and role release may be done, considering that Rule 21 of the HPCSA rules provides that ‘a practitioner shall perform, except in an emergency, only a professional act for which she or he is adequately educated, trained and sufficiently experienced’ (HPCSA 2009). The expectation of shifting towards disease prevention and health promotion is in line with the requirements of the White Paper on the transformation of health in South Africa (DOH 1997).

### Theme 5: Eradicate professional hierarchy

The successful integration of physiotherapists in PHC is said to require the elimination of professional hierarchy amongst health care professionals. Participants shared the same sentiments in this study as per the quotations below:

‘At the district level, physiotherapists must actively take the role of a collaborator because, in these specialised teams, the doctor is always dominating that role. Physios must be a part of the multidisciplinary task team for managing patients … Physios are very good at collaborating and being the liaison person in teams. Multidisciplinary teams lack the ability to look at the biopsychosocial aspects of patients. It is always just treat, discharge. So, I think the physio can advocate in terms of that and look at the quality of life’. (Exp 5, 37 years old, female)

‘Nurses remain the backbone of the South African health system, but we are first-contact practitioners in South Africa; we must manage the patient without interference from any other member of the healthcare team’. (Exp 4, 55 years old, male)

‘We need to move from just being physiotherapists taking advice or prescription from doctors into playing a much more proactive administrative and leadership role … Medical doctors cannot take on the entire role of managing the healthcare system. The roles generally should be shared and shifted … There is an enormous management role for physiotherapists to plan services at PHC or district level. What is the burden of disease in each one of those places? What is the impact of therapy? We can look at cost-effectiveness, analysis, administration, teaching, planning, and evaluating services. Once physios are in administration, they are in a position to push for more resources’. (Exp 7, 64 years old, female)

‘The focus is very much on the primary health care nurse. Therapists do not seem to feature much… They have a big role to collaborate with other services, other government departments, private services, NGOs [*non-governmental organisations*], employment, labour, and education … physios. Have that collaborative framework, and the physios should be part of the team’. (Exp 8, 55 years old, female)

‘We need to expand our roles to involvement in inter-sectorial teams being involved in the department of labour services, transport, and the municipality’. (Exp 9, 44 years old, female)

Carryer (2004) reported that the historical dominance of medical practitioners or doctors had been a significant factor in the tensions and difficulties surrounding the first stage of New Zealand’s PHC implementation as ‘medicine still wishes to see itself as captain of the team’. In contrast, Schäfer et al. (2010) reported that primary care with gatekeeping medical practitioners at the core was a strong foundation of the Dutch healthcare system till 2005. However, in 2006, the Dutch situation changed when physiotherapy became accessible without a referral (Swinkels 2008).

Hierarchies where professional dominance of one discipline over another, as seen in nurses (Ferguson & Anderson 2021), could be why physiotherapists are currently not exercising autonomy within multidisciplinary teams (MDTs). Shared power and leadership might be challenging for physiotherapists within complex traditional hierarchical relationships, particularly those involving physicians or medical practitioners, as Sturm et al. (2022) reported. To dismantle professional hierarchies where medical practitioners and nurses are seen as the default leaders, physiotherapists should be proactive, occupy leadership positions within the MDTs and collaborate with other health care professionals.

### Theme 6: Increase physiotherapy workforce

South Africa has just over 8000 physiotherapists servicing a population of over 60 million people (HPCSA 2021; STATSSA 2021), which is undoubtedly not enough. A study by Ellapen et al. (2019) has suggested that the number of physiotherapists in South Africa is inadequate (1:5667 physiotherapist to patient ratio) to cope with the demand, particularly in the management of patients with non-communicable diseases. The growth in South African physiotherapists is reported to be only 3.4% per year (Ellapen et al. 2019). Increasing the number of physiotherapists in the country should be the aim; however, that takes time. The suggested strategies to strengthen the workforce are based on correcting the urban–rural and private–public divide (see quotations below):

‘To play a major role in a community and healthy environment, we must allocate our human resources to the community … We can place tertiary level physiotherapists at the primary healthcare level to help supervise new physios so that people can gain access to physiotherapy services at that level’. (Exp 1, 42 years old, male)

‘Physios are placed in rural areas where there is a huge need, but these physios are young and alone without formal mentorship or supervision’. (Exp 2, 42 years old, female)

‘I think we need to provide people with more guidance by providing them with mentors’. (Exp 8, 55 years old, female)

‘Allow private practitioners to have contracts with the government and offer services, sort of locum services within the government facilities, because of shortage of health personnel’. (Exp 9, 44 years old, female)

‘I think we need to follow the UK [*United Kingdom*] model, where private and public services collaborate to provide care for everyone’. (Exp 3, 36 years old, female)

‘In the government sector, there is a very limited measure of satisfaction, which might not translate to income for the practitioner. The workload ratio versus the capacity or workforce is the issue’. (Exp 5, 37 years old, female)

A study by Naidoo (2012) found that 84% of the South African population relied on public health services mainly due to affordability, with only 16% using the better and sufficiently funded private health sector. In South Africa, most physiotherapists practice predominantly in urban-based private practice. Some reasons for the unequal distribution of physiotherapists are apartheid segregation, emigration and better working conditions in the private sector (Morris et al. 2021).

To increase the physiotherapy workforce in the PHC, the Department of Health may contract therapists in the private sector to come and do sessions or half days in the PHC centres. Such contracting will ensure that the private physiotherapists will provide the necessary supervision and mentorship to newly qualified therapists and inexperienced therapists in PHC settings, especially in under-resourced areas.

## Limitations

Some of the limitations of our study was a small sample of participants. All participants had the country’s highest educational qualification (PhD), but that does not necessarily make them the most knowledgeable persons in the subject area. Academics have the necessary knowledge but are not working at a PHC level. The authors recommend that future researchers consider having academics and clinical physiotherapists who are in practice and working at related levels of care.

## Implications

The findings of our study may prompt evidence-based practice to quantify and justify physiotherapy’s value in the PHC setting. It is also envisaged that our study’s results may positively impact several areas, such as health policy, undergraduate physiotherapy training, post-graduate training and clinical practice.

## Conclusion

Physiotherapists have a valuable contribution to make to PHC in South Africa, and strategies are required to ensure optimal integration of the profession in the setting. The profession is not well known in South Africa, especially in black and rural communities. As such, something must be done to improve societal knowledge of the profession. There is no point in integrating physiotherapy in PHC if most users of such services have no idea what the profession’s role is. The regulator (PPB) has a role in ensuring that the profession is represented in health policies. They are also empowered in terms of the *Health Professions Act* to advise the Minister of Health on matters pertaining to the general public’s health.

The physiotherapy profession in South Africa mostly operates at a secondary and tertiary level of care and not at PHC levels. Additionally, the current focus is curative and not on disease prevention and health promotion. For this reason, there is a need to further (and quickly) transform the training and education of physiotherapists to focus on disease prevention and health promotion as well as functioning at a primary level of care. The broadening of the role of physiotherapy in South Africa, whether by advancing the scope (Fennelly et al. 2020) or extending it (Bastiaens, Barten & Veenhof 2021; Monteith et al. 2019), should consider the regulator’s ethical rules. Physiotherapists should also be proactive and collaborate with other health care professionals to dismantle the unprogressive professional hierarchy. Suppose the urban–rural and private–public divide is not addressed? In that case, the physiotherapy workforce cannot improve to the detriment of PHC.
